# Spatial patterns and determinants of inter-provincial migration across age groups in China

**DOI:** 10.1371/journal.pone.0330948

**Published:** 2025-08-29

**Authors:** Hua Zhang, Chunyun Chen, Xin Li

**Affiliations:** Beijing Key Laboratory of Environmental Remote Sensing and Digital Cities, Faculty of Geographical Science, Beijing Normal University, Beijing, China; IMT Institute for Advanced Studies Lucca, ITALY

## Abstract

Individuals in different life stages have different intentions and reasons for migration, which leads to differences in the spatial patterns of migration across age groups. This paper aims to reveal the different patterns of inter-provincial migration across age groups and the underlying driving factors to foster a deeper understanding of migration phenomena in China and support an appropriate policy response. Using data from the 2017 China Migrants Dynamic Survey and an extended gravity model with lasso-penalized Poisson regression, we reveal significant heterogeneity in migration patterns among different age groups. In terms of spatial patterns, while people of all age groups tend to migrate from less developed regions to more developed regions, the migration flows of the working-age population are primarily short-distance relocations from populous central provinces to economically developed areas, whereas elderly individuals migrate predominantly from northern regions to Beijing and from southern regions to Shanghai. In terms of influencing factors, while economic considerations drive migration across all age groups, economic opportunities play a significantly stronger role in the working-age population. In contrast, elderly individuals tend to prioritize environmental comfort in their destination choices and are less constrained by distance.

## Introduction

Before the reform and opening-up, labor mobility in China was restricted by the registered residence (*hukou)* system, effectively confining workers to their place of household registration and prohibiting migration. Since the reform and opening-up policies were implemented, these restrictions have been gradually relaxed, allowing laborers from the central and western regions of China to migrate to the eastern coastal areas in search of better employment and development opportunities. This labor migration has been one of the key drivers of the rapid economic growth in eastern China [1]. In 2020, China’s migrant population reached 380 million. In all provinces except Guangxi and Chongqing, the proportion of migrants in the total population exceeded 20.0%, indicating that population mobility has become a widespread phenomenon with significant impacts on the country’s economic and social development. By analyzing the attractiveness of regions to migrant laborers, we can gain insights into the economic development potential of different areas.

Elderly migrants have recently come to represent an increasingly noticeable part of China’s migrants. Therefore, understanding the differences in migration patterns between them and the middle-aged and young migrant population, and providing supporting basic service facilities is a key task in the current aging society. Since China became an aging society, its age structure has changed significantly [2]. In 2020, the number of elderly people over 60 years old reached 264 million. Moreover, an increasing number of seniors in their early 60s are joining the migrant population [3]. In 2020, the proportion of migrants in their 60s reached 12%, approximately 1.5 times higher than the figure in 2010. It is worth noting that the middle-aged and young population still make up the majority of the migrant population, but their driving factors may differ fundamentally from those of the elderly population. This heterogeneity requires research to go beyond isolated analysis of specific age groups and instead conduct comparative analysis of migration characteristics across different age groups to identify the preference distribution and core constraints of population migration in different age groups.

In this paper, we aim to uncover the spatial patterns and driving factors of inter-provincial migration across various age groups in China based on the enhancement of a Poisson gravity model with the integration of a lasso penalty term. It is important to emphasize that our research mainly focuses on the population migration flows between provinces within China, rather than the internal population migration within a province. Our findings support the understanding of the migration dynamics of laborers in different age groups and yield recommendations on how regions can attract laborers from various perspectives. Additionally, this study provides a reference for infrastructure development in different regions during the aging era.

The structure of this paper is as follows. The relevant literature and research progress are summarized in the section of Literature review. The section of Data and methods introduces the data sources and research methodology, focusing on the modified gravity model and the selection of its parameters. The section of Result presents the spatial characteristics of inter-provincial migration across different age groups, and explores the driving factors behind such migration for each age group. Finally, the section of Conclusion presents the conclusions and the implications of the study findings.

## Literature review

Different theories offer explanations of migration from various perspectives. The push and pull factors in migration emphasize that people are “pushed” from their original area of residence while simultaneously being “pulled” toward their new host location [4]. Push factors may include low wages, adverse environmental conditions, political conflicts, etc., whereas pull factors may encompass employment opportunities, education, quality of life at the destination, etc. This theory underscores the importance of considering both the conditions at the destination and the conditions at the origin when exploring migration decision-making. In neoclassical economic theory [5] and new migration theory [6,7], there is a common focus on the key role of economic factors in population migration. According to these theories, individual migrants decide whether to migrate by considering the costs and benefits of different regions on the basis of utility maximization. The cost of living includes mainly housing costs and daily spending, while the benefits can be observed at the per capita wage level of the region. Consumer city theory [8] and new economic geography theory [9] emphasize that comfort and quality of life are not negligible when individuals make decisions on migration. Moreover, empirical studies based on China’s population census show that people’s relocation decisions have become more diverse and that comfort relocation after 2010 cannot be ignored [10]. Comfort includes the convenience of life provided by adequate medical and educational resources, as well as the livability of the natural environment.

The gravity model incorporates origin and destination factors that may affect migration. Since the gravity model has been proven to explain migration fairly well, it is often used in migration studies. The gravity model was originally proposed by Zipf in 1946 [11] and considers only three variables: the distance between the origin and destination and the population sizes of both locations. Scholars subsequently enhanced the model’s explanatory power by incorporating socioeconomic factors from both the origin and destination [12,13]. Another group of scholars has focused on improving the efficiency of estimation and reducing estimation errors of the gravity model itself, proposing a series of models, such as the multilevel gravity model [14], negative binomial Poisson model [15], and Poisson gravity model [16]. However, the issue of multicollinearity among explanatory variables often arises, violating the basic assumption of independence among variables and leading to endogeneity issues and misleading conclusions [17]. The LASSO method proposed by Tibshirani is effective for variable selection, which helps reduce multicollinearity among predictors and thereby improves the model’s fit to the data [18].

Migration has long been a significant topic for scholars in sociology and geography. Migration studies often focus on the total migration volume, trends, influencing factors at different regions and scales, and socioeconomic impacts on both destination and origin areas [19–23]. Since the mid-1980s, there has been a significant migration of rural populations to urban areas in China, while large populations from central regions have moved toward richer eastern regions; this has contributed to the development of towns across the country and the eastern regions in particular [24,25]. Since 2010, with the implementation of the new-type urbanization and western development strategy, migration China has undergone new changes. Spatially, the reduction in the economic gap at the regional level has led to the decentralization of migration destinations. The attractiveness of the central and western regions has increased, and there is a trend of return migration from the eastern developed regions to the central and western regions [26,27]. In terms of influencing factors, along with improvements in people’s living standards, factors such as the level of regional public service and environmental comfort are gradually exerting stronger influence on migration. In this new development stage, migration in China exhibits increased complexity. The enhanced attractiveness of the central and western regions may improve their economic development in the future.

Migration characteristics vary significantly across age groups, reflected in both the frequency and purpose of migration. According to life course theory [28], individuals face different needs and opportunities at different life stages, shaping their migration behavior. Rogers proposed the classic “age-specific migration rate” model, showing that migration rates peak during the prime working-age years, with a smaller peak around age 60–70 [29]. The capability–aspiration–opportunity framework further elucidates these differences by emphasizing that migration depends not only on individuals’ desire to move, but also on their capacity and the opportunities available to do so—each of which varies considerably across age groups [30]. In China, there is existing literature that treats the population migration as a whole and examines the heterogeneity in their destination choices and underlying motivations [31,32]. However, discussions specifically focusing on the heterogeneity of spatial patterns and determinants of migration across different age groups—which is the main concern of this study—are relatively limited. Existing studies primarily focus on differences in migration intensity among age groups. Research has found that younger cohorts tend to exhibit stronger migration intentions [33,34], and the age distribution of China’s population migration generally peaks around age 27 and then gradually declines [35]. It is worth noting that although existing research has analyzed the reasons for migration reported in census-based surveys using statistical methods [36], the mechanisms underlying the spatial heterogeneity of migration across age groups remain underexplored. In summary, while current studies have shed light on the intensity and reasons behind the age patterns of China’s population migration, more in-depth research is needed to uncover the spatial heterogeneity of migration among different age groups.

The varying attractiveness of different regions to different age groups has far-reaching implications for their subsequent development. By examining the heterogeneity of migration across different age groups, we can obtain a clearer picture of migration patterns across different age groups amidst changing migration dynamics. Additionally, with the arrival of an aging society, regions that aim to attract elderly individuals need to undertake comprehensive planning and construction of supportive infrastructure to meet the needs of the aging population.

## Data and methods

### Data

The research area of this paper covers 31 provincial-level administrative units in mainland China. The population data are sourced from the 2017 China Migrants Dynamic Survey (CMDS), a nationally representative, large-scale sample survey conducted by official institutions. The CMDS targets migrants over 15 years old who have the ability to migrate independently and have resided in their current location for more than one month but do not have local (county or city) household registration. The data are provided by the China Population and Development Research Center, with key fields selected for analysis, including year of birth, current residence, and registered domicile (hukou). These data exclude transient populations at locations such as stations, docks, airports and hospitals and student migrants.

Owing to the significant differences between the number of sampled migrants and the actual migrant population in each province, we adjust the migrant population of each migration flow by multiplying it by the ratio of the number of individuals whose current residence differs from their registered household location in the seventh national census to the number of sampled migrants in the 2017 China Migrants Dynamic Survey. This reweighting approach aims to correct for potential sampling bias in the CMDS data and to approximate the actual scale of inter-provincial migration flows. Similar proportional adjustment methods have been used in previous migration studies to reconcile differences between sample surveys and population-level data [37,38]. Since the CMDS is a large-scale sample survey but does not provide province-level expansion weights, using the census as a benchmark helps enhance the representativeness of the data at the provincial scale. After data weighted adjustment, the total number of migrants in each province is basically consistent with the number of migrants entering each province in the seventh national census. According to the United Nations, individuals aged 15–29 years are classified as youth, those aged 45 years and above are considered middle-aged, and those aged 60 years and above are classified as elderly. On the basis of the United Nations definition and China’s national context [39], we classify inter-provincial migrants into four categories: young migrants aged 15–29 years, early middle-aged migrants aged 30–44 years, middle-aged migrants aged 45–59 years, and elderly migrants aged 60 years and above. After selection, the total number of valid inter-provincial migrant samples is 83,793, including 24,965 valid samples of young migrants, 38,166 samples of early middle-aged migrants, 18,114 samples of middle-aged migrants, and 2,548 samples of elderly migrants.

The socioeconomic data are from the China Statistical Yearbook 2018 and the statistical yearbooks of various provinces in 2018. The shortest railway distance between provincial capitals is calculated on the basis of the original railway data of China from the OpenStreetMap website using ArcGIS. The PM2.5 values for the environmental indicators are sourced from the Atmospheric Composition Analysis Group, and the temperature data for each province are sourced from the China Meteorological Data Service Centre.

### Variable selection

As shown in the literature review, migration can be influenced by six key factors: distance, population size, regional economic level, social development level, cost of living, and environmental comfort. We select 10 explanatory variables in these six dimensions. Although it is not possible to capture all potential influencing factors, the six dimensions included have been shown in previous research to possess strong explanatory power and are closely aligned with the central theme of this paper—namely, what types of regional characteristics are more attractive to populations of different age groups. Additionally, certain factors that are theoretically important, such as social networks, were excluded due to difficulties in data acquisition and their relatively limited relevance to the research focus. The descriptive statistical characteristics of the explanatory variables are shown in Table 1.

**Table 1:** Descriptive statistical analysis of variables.

Variable Type	Variable	Description	Mean	Min	Max
Explained variable	M_ij_	Number of inter-provincial migrants by age group in 2017 (persons, in natural logarithm)	8.61	2.08	12.00
Population size Distance	POP_i_ /POP_j_	Resident population at census points in the province in 2017 (10,000 people, in natural logarithm)	8.14	5.86	9.40
	D_ij_	Shortest railway distance between provincial capitals in 2020 (kilometers, in natural logarithm)	7.22	5.25	8.29
Regional economic development level	GDP_i_ /GDP_j_	GDP growth rate of the province in 2017 (%)	7.21	3.40	10.20
	WAG_i_ /WA_j_	Average annual wage of on-post urban unit employees in the province in 2017 (yuan, in natural logarithm)	11.21	10.93	11.81
Regional social development level	DOC_i_ /DOC_j_	Number of physicians per 1,000 people in the province in 2017	6.59	4.90	11.33
	EDU_i_ /EDU_j_	Number of higher education teachers per 10,000 people in the province in 2017	12.38	7.11	31.77
Regional cost of living	HOU_i_ /HOU_j_	Ratio of residential sales price to disposable income in the province in 2017 (%)	0.32	0.18	0.56
	CPI_i_ /CPI_j_	Consumer price index of the province in 2017 (%)	101.58	100.91	102.85
Regional environmental comfort level	AIR_I_ /AIR_j_	Annual average PM2.5 concentration of the province in 2017 (μg/m³)	44.08	17.90	67.60
	TEM_i_ /TEM_j_	Average temperature difference between January and July of the province in 2017 (°C)	25.37	7.17	42.45

The variables reflecting population size, which influences the potential migrant population, include the populations of the origin province (POP_i_) and the destination province (POP_j_). The difficulty of inter-provincial population migration can be indicated by distance, and the shortest railway distance between provincial capitals (D_ij_) is used in this paper. The use of railway distance as a proxy for migration cost is based on its approximation of actual travel accessibility between provincial capitals, particularly in China, where the railway network plays a central role in long-distance migration [40,41]. The regional economic development level is the main factor influencing inter-provincial population migration related variables include the average annual wage of urban employees (WAG_i_/WAG_j_) and the GDP of the origin and destination provinces in 2017 (GDP_i_/GDP_j_). The level of regional social development is a potential factor affecting migration choices, and the quantity and quality of infrastructure are significant aspects. We use the average number of practicing physicians per thousand people (DOC_i_/DOC_j_) and the average number of higher education teachers per ten thousand people in the origin and destination provinces (EDU_i_/EDU_j_) to measure regional social development. The cost of living is increasingly becoming an important factor influencing inter-provincial population migration. The ratio of housing sales prices to the disposable income of residents (HOU_i_/HOU_j_) and the price level index (CPI_i_/CPI_j_) are used to characterize the influence of housing costs and price levels on population migration. With economic development, people pay more attention to quality of life, and environmental comfort has become a new factor of concern. Environmental comfort specifically includes the average PM2.5 concentration in the origin/destination province during the year (AIR_i_/AIR_j_) and the average temperature difference between January and July within the origin/destination province (TEM_i_/TEM_j_).

### Lasso-penalized Poisson gravity model

The gravity model, an important model in migration studies, suggests that the migration between two locations is directly proportional to the populations of the two locations and inversely proportional to the distance between them [42]. In studies of the gravity model, scholars have found that Poisson regression can better address heteroscedasticity issues in linear regression models and provides a better fit for migration flows in reality. Building upon the basic gravity model, we utilize aggregated data at the provincial level and employ the Poisson gravity model. The basic model is formulated as follows.


$$M_{\text{ij}}=\text{exp }(\beta \text{0lnDij }+\sum_{\text{q}=\text{1}}^{\text{10}}\beta_{\text{iq}}\text{ln}{\text{X}}_{\text{i}}+\sum_{\text{q}=\text{1}}^{\text{10}}\beta_{\text{jq}}\text{ln}{\text{X}}_{\text{j}})\mathrm{\backslash ]}$$

where M_ij_ represents the number of migrants from location i to location j within a specific age group, D_ij_ denotes the shortest railway distance between locations i and j, X_i_ represents the relevant indicators of the origin location, Xj signifies the relevant indicators of the destination location. β_0_, β_iq_ and β_jq_ are the coefficients to be estimated.

To improve model parsimony and perform effective variable selection, we apply the LASSO (Least Absolute Shrinkage and Selection Operator) method within the Poisson regression framework. The LASSO estimator is obtained by maximizing the penalized log-likelihood, which imposes an L1 penalty on the absolute values of the regression coefficients. This penalty term encourages sparsity by shrinking some coefficients exactly to zero, effectively performing variable selection. This approach reduces variance inflation and stabilizes coefficient estimates. We then performed Variance Inflation Factor (VIF) tests on the selected variables in each model, and each variable exhibited a VIF value below 5, indicating that multicollinearity is not a significant concern in the final model. Thus, the LASSO method primarily facilitates variable selection, helping improve model interpretability and generalizability while maintaining the essential structure of the data. The basic model is formulated as follows.


β^= argmaxβ[∑i=1n(yijln(Mij)−λi−ln(Mij!))−λ∑j=110|βj|)]

where n is the sample size $\lambda_{\text{i }}=\;exp\;(\beta \text{0lnDij }+\sum_{q=1}^{10}\beta_{iq}lnX_{i}+\sum_{q=1}^{10}\beta_{jq}lnX_{j}),$ λ is the penalty parameter of the L1 regularization term, and the optimal value can be determined through K-fold cross-validation. In this work, the value of λ that minimizes the standard error of the regression predictions in the cross-validation is selected as the final parameter. The Poisson regression, lasso variable selection, and cross-validation methods used in this paper are all implemented using the glmnet package in R language [43,44].

## Result

### Spatial patterns of inter-provincial migration across age groups

Migration propensity generally declines with age, reflected in both the scale and likelihood of migration as individuals grow older. Young and early middle-aged migrants make up the main group of migrants. Young and early middle-aged migrants account for 45.55% and 29.79% of the total migrant population, respectively (Table 2). Similarly, these two age groups have the highest likelihood of migrating to provinces outside their hometown, with 15.53% of young adults and 17.98% of early middle-aged adults engaged in inter-provincial migration. As individuals transition into older age groups, their mobility decreases, with the proportion of middle-aged migrants dropping to 8.01%. By the time individuals reach the elderly stage, mobility significantly diminishes, with elderly migrants accounting for only 1.44% of the elderly population. This suggests that migration is more common among early middle-aged and young people, with the likelihood decreasing with age. This aligns with lifecycle theory, which posits that young and middle-aged individuals are more likely to migrate due to greater opportunities and needs. As individuals enter the elderly stage, opportunities typically decrease, and stability becomes a greater concern, resulting in significantly reduced mobility.

**Table 2:** Inter-provincial migration by age group.

Age groups	Inter-provincial migrants (thousands)	Share of total migrants	Inter-provincial Migration Rate within Age Group
15–29	37,194	29.79%	15.53%
30–44	56,869	45.55%	17.98%
45–60	26,987	21.62%	8.01%
>60	3,796	3.04%	1.44%
All	124,837	100%	10.80%

#### Spatial pattern of in-migration.

**Fig 1:**
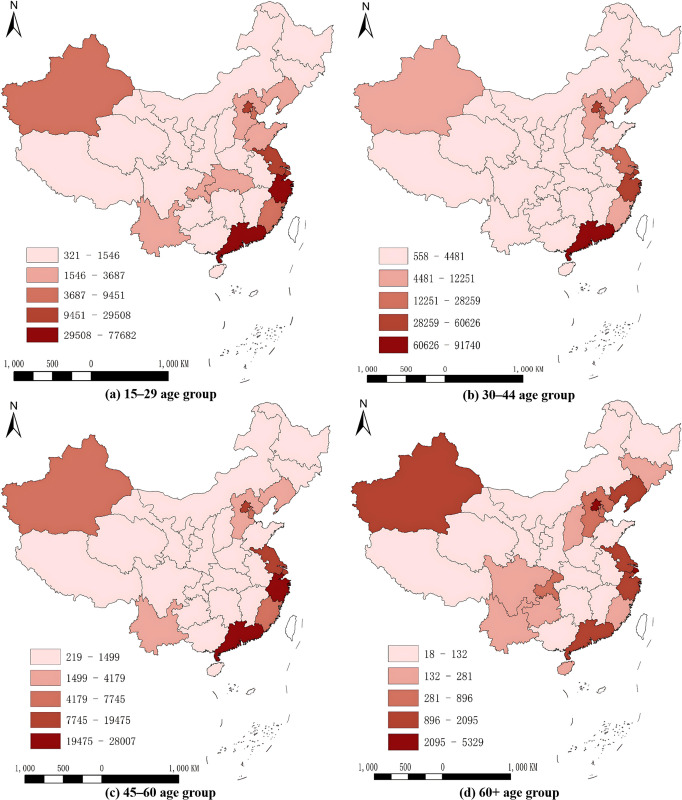
Distribution of the number of incoming migrants by province. Note: The map was created by the authors using ArcGIS 10.8, based on the standard national map (Review Number: GS(2020)4630) provided by the Ministry of Natural Resources of the People’s Republic of China (http://bzdt.ch.mnr.gov.cn/).

Overall, migrants tend to migrate to economically developed regions. Five provinces, including Guangdong, Zhejiang, Shanghai, Beijing, and Jiangsu, are the top choices for inter-provincial migrants (Fig 1), accounting for 24.78%, 17.51%, 12.98%, 10.43%, and 5.85%, respectively. Except for Jiangsu, these regions are preferred destinations for migrants across all age groups. The developed industries, high welfare, healthcare, and education of these areas contribute to their attractiveness as destinations for inter-provincial migrants seeking better career and life opportunities.

Young migrants exhibit a greater degree of concentration in destination selection, with the top four destinations attracting 68.73% of inter-provincial migrants (Table 3). Among them, Guangdong, the top destination, attracts approximately 28.83% of migrants, whereas the bottom three provinces attract only 0.51% of migrants. However, the bottom three provinces do attract some of the elderly population, with 15.60% of elderly migrants choosing these regions, a relatively higher proportion compared to other age groups. This is because young adults typically seek career development opportunities and thus tend to choose economically developed areas, avoiding economically underdeveloped regions. For the elderly population, areas with high social and economic development are attractive, but they also prefer to migrate to less developed areas with better environmental conditions, such that their destination choices are more diverse than those of other age groups.

**Table 3:** Characteristics of the spatial distribution of the immigrant population by age group.

Age group	Share of the top four provinces	Share of the first province	Share of the last three provinces
15–29	68.73%	28.83%	0.51%
30–44	65.69%	24.85%	0.60%
45–60	58.22%	18.75%	0.70%
>60	61.80%	25.39%	15.60%
All	65.50%	24.30%	0.60%

Migration preferences vary by age group, with distinct regional trends. Youth and early middle-aged migrants predominantly choose the Yangtze River Delta and Pearl River Delta, accounting for 69.60% and 63.24% of their total migration flows, respectively. Middle-aged migrants tend to favor the Yangtze River Delta, with 41.25% settling there. Elderly migrants, on the other hand, show almost equal preferences for the Yangtze River Delta, at 32.20%, and the Beijing**–**Tianjin**–**Hebei region, at 32.17%, which may reflect the unique appeal of these areas to this age group. These trends highlight differing priorities: young and middle-aged migrants may be motivated by the economic opportunities and career prospects in the Yangtze River Delta and Pearl River Delta, whereas elderly migrants tend to value stability, the social environment, and access to quality healthcare, making both the Yangtze River Delta and Beijing**–**Tianjin**–**Hebei regions potentially attractive.

#### Spatial pattern of out-migration.

In general, the outflow population originates from four populous provinces in central China, namely, Anhui, Henan, Sichuan, and Hunan; migrants from these provinces account for 40.13% of the total outflow population (Table 4). Data of various age groups show that the outflow population from these four provinces accounts for 43.81%, 39.93%, 45.78%, and 33.78% of the total young, early middle-aged, middle-aged, and elderly migrant populations, respectively. These findings suggest that these provinces are major sources of the country’s labor force.

**Table 4:** Characteristics of the spatial distribution of the emigrating population by age group.

Age group	Share of the top four provinces	Share of the top province	Share of the last three provinces
15–29	43.81%	14.42%	0.09%
30–44	39.93%	13.03%	0.25%
45–60	45.78%	16.10%	0.18%
>60	33.78%	9.81%	0.44%
all	40.13%	13.93%	0.22%

**Fig 2:**
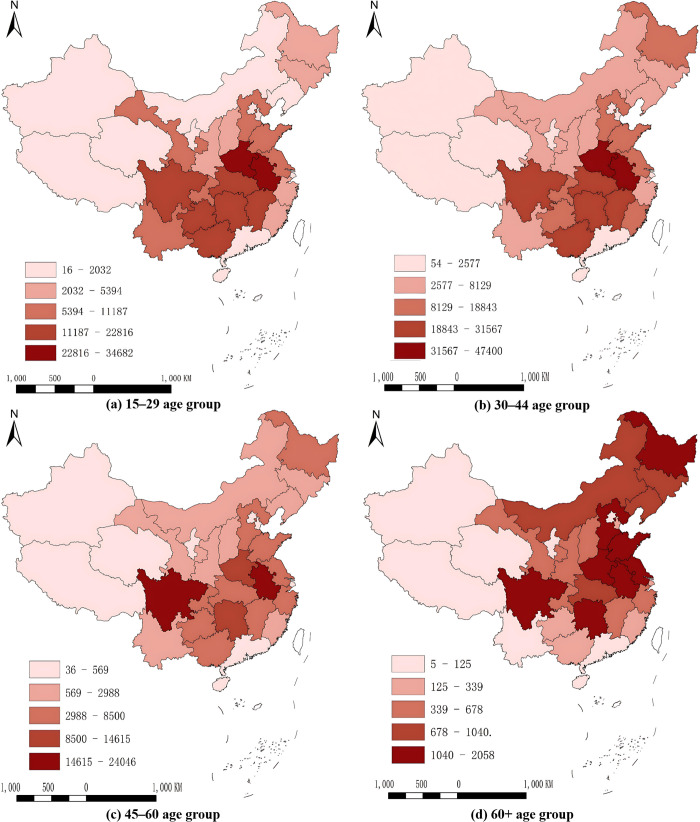
Distribution of the number of outgoing migrants by province. Note: The map was created by the authors using ArcGIS 10.8, based on the standard national map (Review Number: GS(2020)4630) provided by the Ministry of Natural Resources of the People’s Republic of China (http://bzdt.ch.mnr.gov.cn/).

The mobility of the elderly population in northern regions appears to have increased noticeably, with Northeast China experiencing a marked increase (Fig 2). Specifically, the outflow population from northern regions constitutes 30.81%, 32.98%, 30.57%, and 51.72% of the total outflow population in the young, early middle-aged, middle-aged, and elderly groups, respectively. Notably, the migration rate of elderly people is more than 20 percentage points higher than that of middle-aged people, jumping from 30.57% to 51.72%.

This trend appears to be more evident in Northeast China, where elderly migrants represent 17.51% of the total elderly outflow nationwide, even though the region contributes only 5.44% of the overall national outflow population across all age groups. Among the provinces, Heilongjiang has shown a noticeable rise in elderly outflow, becoming the largest source of elderly migrants. Similarly, Liaoning and Jilin have experienced significant elderly migration, ranking among the top ten regions with the largest outflow populations.

This pattern may be associated with the unique needs of elderly individuals, who generally require more favorable environmental conditions than individuals in other age groups do. The harsh climates of northern regions, particularly those characterized by severe cold during the winter, may be less suitable for elderly people. As a result, many elderly individuals choose to relocate to areas with warmer climates and more livable environments.

#### Spatial patterns of migration flows.

**Fig 3:**
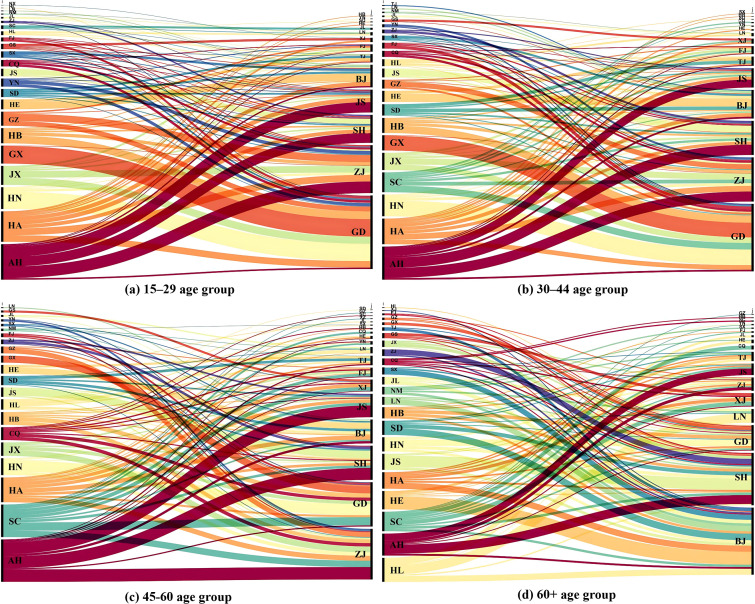
Top 100 migration flows by age group. Note: The labels on the diagram denote provincial abbreviations based on the initials of their pinyin spellings. For example, GD stands for Guangdong province.

When choosing migration destinations, individuals across all age groups tend to prioritize proximity, favoring economically developed regions nearby. For example, residents from Anhui, a major source of the outflow population, migrate predominantly to Zhejiang, Jiangsu, and Shanghai (Fig 3), with these destinations accounting for 19.39%, 26.48%, and 28.70% of Anhui’s migrant population, respectively. Similarly, migrants from Guangxi and Hunan overwhelmingly choose Guangdong, with 73.26% of Guangxi’s emigrants and 65.70% of Hunan’s emigrants migrating to Guangdong. Proximity often plays a significant role in migration decisions, as shorter distances entail reduced costs and easier adaptation, and cultural and linguistic similarities between neighboring regions facilitate integration.

The majority of migration flows among working-age groups originate from populous central provinces, with routes such as Guangxi–Guangdong, Hunan–Guangdong, Anhui–Zhejiang, Anhui–Shanghai, Anhui–Jiangsu, Sichuan–Guangdong, and Hebei–Beijing ranking among the top ten. For working-age individuals, the primary motivation is to secure a job and opportunities; thus, economically developed regions with ample employment and convenient transport are key destinations for migrants from central provinces with abundant labor.

The main migration flows of elderly migrants are from northern regions to Beijing and from southern regions to Shanghai, which present a noticeable contrast with the migration patterns of working-age groups. Among the top twenty migration flows, six involve migrants from northern regions migrating to Beijing, and five involve migrants from southern regions migrating to Shanghai. These two cities attract the largest number of elderly migrants, whereas Guangdong and Zhejiang are favored by younger age groups. This is primarily because one major reason for elderly migration may be care for grandchildren, with roughly one-quarter of elderly migrants relocating for this purpose. Young migrants working in Beijing and Shanghai face significant pressures in daily life and may frequently rely on their parents for childcare support. As a result, Beijing and Shanghai have become key destinations for elderly migration.

### Determinants of inter-provincial migration across age groups

#### Model selection.

In this study, we construct lasso-penalized Poisson gravity models to identify the factors influencing inter-provincial migration across different age groups. The results reveal significant multicollinearity among the independent variables in the Poisson gravity model, with VIF values exceeding 10. In contrast, the use of the lasso-penalized Poisson gravity model successfully mitigates this issue, reducing the VIF values of all independent variables to below 5. This improvement may lead to a more accurate estimation of the independent variables and enhances the model’s explanatory ability. To ensure the appropriateness of comparing migration flows across different age groups, we also conducted a variance test (Bootstrap) on the migration flow data. The test results indicate no significant differences in variances between groups (p > 0.05), supporting the comparability of these datasets in a unified modeling framework. Additionally, using the bootstrap method, we calculated the p-values for each independent variable and obtained their significance levels, further validating the robustness of our analysis. Moreover, the lasso-penalized Poisson model demonstrates strong performance, with R² values for all age groups exceeding 0.68, indicating a good fit to the migration flow data (Table 5). These findings demonstrate that the lasso-penalized Poisson gravity model provides a robust and reliable representation of real-world migration patterns.

**Table 5:** Results of lasso-penalized Poisson model regression across age groups.

Variables	Migrants aged 15–29	Migrants aged 30–44	Migrants aged 45–59	Migrants aged 60+	All migrants
POPi	0.76***	0.80**	1.00***	0.88***	0.88***
POPj Dij	1.6***	1.59***	1.01***	0.95***	1.57***
	−1.28***	−1.22***	−1.1***	−1.08***	−1.20***
WAGi	−4.26***	−3.52***	−2.54***	−1.80**	−3.23***
WAGj	4.58***	4.28***	4.08***	3.89***	4.20***
GDPi	0.17***	0.03***	0.27***	0.14***	0.10***
GDPj	−0.36***	−0.08***	−0.23***	–	−0.24***
HOUi	0.19***	−0.61***	−0.26**	0.71***	−0.26***
HOUj	3.38***	6.02***	2.89***	1.33***	4.59***
CPIi	−0.55***	−0.33***	−0.49***	−0.29***	−0.45**
CPIj	0.91***	1.19***	0.83***	0.67***	0.99***
DOCi	−0.2***	−0.23***	−0.27***	−0.19***	−0.23***
DOCj	0.29***	0.03***	0.3***	0.04***	0.14***
EDUi	–	0.03***	0.04***	0.05*	0.03**
EDUj	−0.11***	−0.08***	−0.14***	–	−0.09**
AIRi	−0.01***	−0.01***	−0.02***	−0.03***	−0.02***
AIRj	−0.04***	−0.05***	−0.02***	−0.04***	−0.04***
TEMi	–	0.02***	0.03***	0.07***	0.03**
TEMj	−0.01***	0.04***	0.02***	0.03***	0.03***
Pseudo R²	0.78	0.78	0.68	0.68	0.80

Notes: (1) The lasso penalty excludes variables that fail validation, marked as “-”.

(2) Xi represents the relevant indicators of the origin location, Xj signifies the relevant indicators of the destination location.

(3) *p < 0.1. **p < 0.05. ***p < 0.01.

#### Factors influencing inter-provincial migration.

Population size and distance significantly influence inter-provincial migration. Large provincial population sizes are associated with high migration inflows and outflows, whereas large railway distances between regions reduce migration. These findings highlight the enduring importance of population size in driving migration and the persistent distance decay effect, even as transportation infrastructure continues to improve.

The level of regional economic development significantly influences population migration, with average annual wages being a key factor. Across all age groups, higher wages attract more inbound migrants and reduce outbound migration, reflecting people’s pursuit of better economic opportunities. Additionally, GDP growth rates impact migration, with rapidly growing regions being less attractive to migrants than economically developed areas with slower growth. These findings suggest that people prioritize stable economic conditions and higher income prospects when making migration decisions.

Housing costs play a significant role in shaping migration patterns. Across all age groups, higher housing prices relative to local income levels tend to attract more inbound migrants while slightly reducing outbound migration. Despite the rapid rise in housing prices in recent years, housing costs in 2017 did not diminish people’s willingness to move to developed areas. This suggests that may still be willing to accept higher living expenses in exchange for improved economic prospects and opportunities.

Environmental factors, such as air quality and temperature differences, play a role in shaping migration patterns. Poor air quality, exemplified by high PM2.5 concentrations, contributes to an increase in outbound migration, as severe weather conditions, such as smog, reduce residents’ comfort. Similarly, regions with large temperature differences between winter and summer are less appealing, prompting many people to move away. These findings highlight the growing impact of environmental conditions on population migration decisions in recent years.

### Differences in factors influencing migration across age groups

Regions with large populations are more attractive to young people than to elderly people. For young and early middle-aged individuals in their prime working years, populous regions are particularly appealing because of their abundant job opportunities, educational resources, and vibrant lifestyles. However, as people age, the importance of these factors diminishes, leading to a noticeable decline in the attractiveness of such regions. Elderly people are the group least inclined to migrate to densely populated areas, reflecting their shifting priorities and preferences at later life stages.

As individuals age, the influence of distance on migration decisions gradually diminishes. Young and early middle-aged individuals, who often may bear responsibilities such as family care and career development, tend to face restrictions regarding distance and prefer closer destinations that allow them to balance work and family obligations. In contrast, elderly individuals are less constrained by distance, as their migration decisions are less influenced by family responsibilities and may instead focus on personal preferences, such as lifestyle or living environment. This shift underscores how the life stage and responsibilities shape the role of distance in migration choices.

Wage level has a much stronger influence on migration decisions for young individuals than for elderly individuals. Low wages can push these groups to leave, whereas high wages can be highly attractive. Young adults, who may prioritize career development and financial independence, are especially sensitive to changes in wage levels, with the pull of low-wage regions diminishing significantly as age increases. Similarly, high wages remain attractive across all age groups but are slightly less impactful for middle-aged and elderly individuals, who may place greater emphasis on other factors.

High housing costs in one’s hometown act as a push factor for young and elderly populations but as a pull factor for early middle-aged and middle-aged groups. For young people, who are often in the early stages of their careers and have unstable finances, the economic burden of purchasing a home may motivates them to leave high-cost regions. Similarly, elderly individuals, influenced by a culture of frugality and a fixed income, are likely to migrate away from areas with expensive housing. In contrast, early middle-aged and middle-aged individuals, typically in their peak earning years, may view high housing costs as indicators of stronger economic opportunities and social benefits, which makes such regions more attractive to them. This divergence reflects how housing costs interact with age-specific financial priorities and migration motivations.

Elderly individuals are attracted to regions with moderate climates and good air quality. The cold northern climate, characterized by large temperature differences, acts as a significant push factor for elderly individuals, as they are less comfortable in such harsh climates. These migrants tend to prefer warmer areas. Additionally, air quality is an important consideration for elderly individuals, and good air quality serves as a pull factor. These findings suggest that, when choosing migration destinations, older individuals may prefer comfortable regions for healthy concern.

### Mechanism of inter-provincial migration across age groups

**Fig 4:**
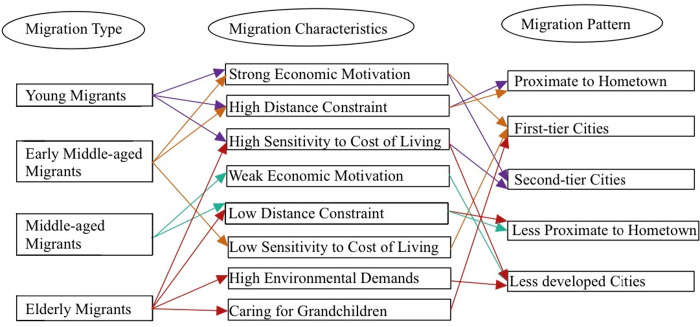
Diagram of migration mechanisms by age group.

Migration patterns vary across age groups, with each group exhibiting distinct characteristics (Fig 4). For young migrants, economic factors represent the primary driver of migration. However, these individuals’ limited capital and low migration flexibility constrain their options. Distance is a major barrier for this group, and as members of the one-child generation, family considerations may lead to more conservative choices. While they tend to leave low-income areas in favor of economically developed regions, high living costs limit their mobility. Consequently, they often choose destinations close to their hometowns that offer both economic opportunities and affordable housing and avoid high-cost cities, such as Beijing, Shanghai, and Shenzhen.

Early middle-aged migrants are also economically driven but have sufficient capital accumulation and relatively low migration flexibility. Although they are driven by economic factors, their better economic conditions result in fewer living cost constraints than young individuals with regard to destination choices. Additionally, being sandwiched between caring for older and younger family members, they tend to migrate to nearby areas.

Middle-aged migrants have some capital accumulation and greater migration flexibility. As they age, their mobility decreases significantly. Their desire for better development opportunities weakens, and the attractiveness of developed regions might diminish. With reduced family caregiving burdens, their migration flexibility increases.

The mobility characteristics of elderly populations are shaped by concerns such as environmental factors, caregiving responsibilities, migration freedom, and economic thriftiness. Environmental considerations, including air quality and temperature suitability, play a key role in their migration decisions. For example, elderly populations in northern regions tend to be more mobile than their counterparts in southern regions, and they often seek warmer climates. About 19.60% of elderly migrants move to care for their grandchildren — which may represent a significant driver of migration, especially in cities like Beijing and Shanghai, where young parents face high childcare pressures and depend heavily on parental support. Additionally, elderly individuals enjoy greater freedom from family and work obligations, such that they are little affected by distance constraints. However, economic thriftiness remains a notable factor, as they prioritize destinations with low living costs.

## Conclusion

In this paper, we employ a lasso-penalized Poisson model to analyze inter-provincial migration patterns across different age groups in China. While many studies address migration flows as a whole, with a focus on general migration patterns within and across regions or on rural–urban divisions, they often neglect to distinguish migration choices according to age-specific factors. By addressing these differences, this study provides a detailed examination of migration patterns across age groups, which represents a key academic contribution of this paper.

This paper yields the following conclusions. First, young and middle-aged migrants prefer the Yangtze River Delta and Pearl River Delta regions as migration destinations, while middle-aged and elderly populations prefer the Yangtze River Delta region, and elderly populations tend to migrate to the Yangtze River Delta and Beijing–Tianjin–Hebei regions.

Second, outbound migration among the working-age group originates mainly from the populous central provinces, with most migrants moving to nearby economically developed areas. In contrast, outbound migration among elderly populations originates mainly from northern regions, especially the northeast region, with migration to Beijing predominating.

Third, regions with large populations strongly attract young people but not elderly people. Young people are most constrained by distance, whereas elderly people are least affected. Wage levels are key for young and middle-aged migrants, whereas elderly people prioritize environmental comfort. High hometown housing costs drive migration among thrifty young and elderly groups, whereas middle-aged migrants are drawn to areas with high housing costs. These factors shape distinct migration patterns across age groups.

The paper provides key policy recommendations. First, local governments should prioritize talent retention policies by expanding housing support and adaptation subsidies for young migrants. Migration among the young population is strongly driven by economic factors, but long distances and high housing costs act as push factors. As a result, young people tend to choose nearby first- or second-tier cities with good economic development rather than focusing on first-tier cities. This creates significant development opportunities for these regions. Local governments should prioritize talent retention measures in their policies. To attract and retain high-skilled youth, local governments should improve rental housing options and offer targeted subsidies that help young migrants adapt to new environments and build a sense of belonging.

Second, governments in developed regions should implement comprehensive family support policies for middle-aged migrants. Developed areas like the Yangtze River Delta and Pearl River Delta attract many early middle-aged and middle-aged migrants who often relocate with families. To retain this population and ensure long-term social stability, local authorities should enhance access to quality education for children and improve social welfare, including healthcare and social security systems, tailored to the needs of migrant families.

Third, local governments should strengthen cross-regional healthcare systems and eldercare infrastructure to meet the needs of elderly migrants. Cities such as Beijing and Shanghai attract a significant number of elderly migrants from other regions. Cities such as Beijing and Shanghai host a growing number of elderly migrants. These individuals require not only age-friendly urban environments but also access to continuous healthcare services. Establishing a cross-regional medical insurance system is essential to ensure treatment continuity for mobile seniors. Additionally, as elderly migrants often move to cities with better environments but are limited by costs, governments should improve healthcare services in smaller, attractive cities to support healthy aging and ease pressure on megacities.

Owing to limitations in the data at the city scale, we focus on migration patterns at the provincial level without delving into more detailed subdivisions. Within the same age group, there are significant variations, such as differences in migration preferences between urban and rural populations, as well as variations in education level and occupation. These aspects represent areas that warrant further exploration in future research.
